# Supplementary material for: Scaling-up molecular logic to meso-systems via self-assembly
Source: Nat Commun. 2025 Mar 28;16:3015. doi: 10.1038/s41467-025-58379-0 (PMC11950191; doi:10.1038/s41467-025-58379-0)
Supplement: Supplementary file 1 — Supplementary Information [file 41467_2025_58379_MOESM1_ESM.pdf]

Scaling-up Molecular Logic to Meso-systems via Self-assembly

Ze-Qing Chen, Brian Daly, Chao-Yi Yao, Hannah S.N. Crory, Yikai Xu, Ziwei Ye, H.Q. Nimal Gunaratne, Ayumi Kimura, Seiichi Uchiyama, Steven E.J. Bell, Eric V. Anslyn, and A. Prasanna de Silva

Supplementary Information

Supplementary methods 1. Synthesis scheme, preparative procedures and characterizations of the principal cyclophanes. Page 2
Supplementary note 1. Spectral characterization for **1**, **4** and **3**. Page 5
Supplementary methods 2. Synthesis scheme, preparative procedures and characterizations of the ancillary cyclophanes and other compounds. Page 11
Supplementary note 2. Photographs of aqueous solutions formed by various mixtures of **1** and **10**. Page 15
Supplementary note 3. Dynamic light scattering (DLS) data. Page 16
Supplementary note 4. Nanoparticle tracking analysis (NTA) data. Page 19
Supplementary note 5. Aggregation of detergents with/without cyclophanes. Page 22
Supplementary note 6. Transmission electron microscopy (TEM) images (enlarged). Page 23
Supplementary note 7. ^{1}H NMR spectral testing of the binding abilities of hosts **4**, **3** and **1** with guest **9**. Page 26
Supplementary note 8. Effect of NaCl on the turbidity of mixtures of cyclophanes **1**, **3** or **4** and detergent **10**. Page 29
Supplementary note 9. Logic gate array displayed by the turbidity output of the cyclophane (**1**, **3** or **4**) – detergent **10** system when xylyldiammonium cation **9** is absent. Page 30
Supplementary references. Page 31

Supplementary methods 1. Synthesis scheme, preparative procedures and characterizations of the principal cyclophanes.

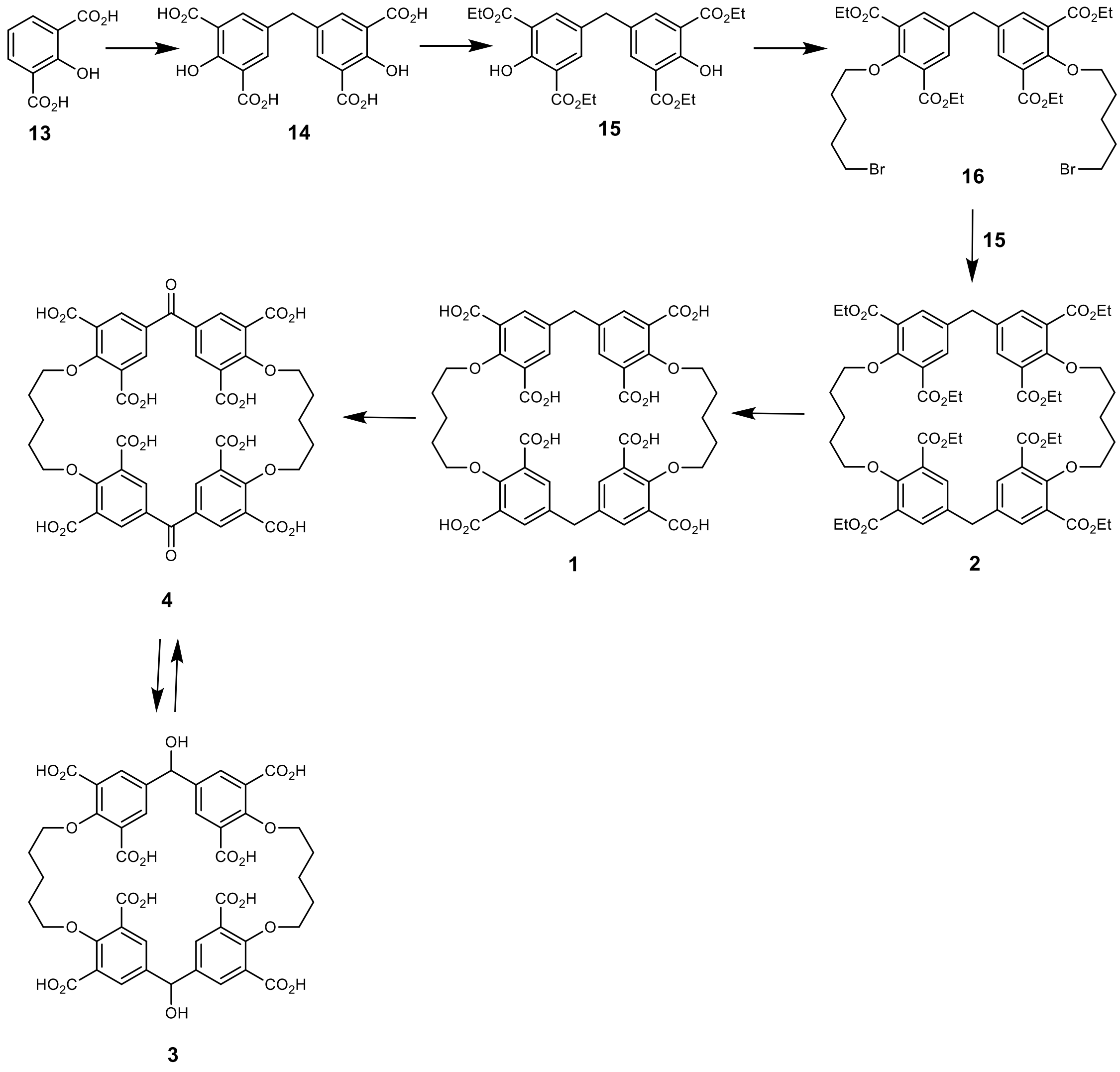

Supplementary Fig. 1. Scheme for the synthesis of cyclophanes **1**, **4** and **3**.

Preparative Procedures

5,5'-Methylenebis(2-hydroxyisophthalic acid) (**14**).

2-hydroxyisophthalic acid, **13**[1], (11 g, 6.04 x 10^{-2} mol) and 98% sulfuric acid (5.92 g, 6.04 x 10^{-2} mol) was refluxed in glacial acetic acid (80 ml) before 37% formaldehyde solution (2.75 g, 3.38 x 10^{-2} mol) was added. The reaction was stirred for 72 hours, before pouring onto crushed ice. The mixture was filtered and dried to yield a cream solid (10.92 g, 48 %).

Melting point = 282 – 283 °C

^{1}H NMR (400 MHz, DMSO-d_6, δ, ppm): 7.80 (s, 4H, Ar<u>H</u>), 3.89 (s, 2H, Ar-C<u>H$_2$</u>).

^{13}C NMR (100 MHz, DMSO-d_6, δ, ppm): 168.96 (<u>C</u>-OOH), 160.36 (Ar<u>C</u>-COOH), 135.43 (Ar-<u>C</u>H), 130.34 (Ar<u>C</u>-CH$_2$), 117.20 (Ar<u>C</u>-OH), 37.96 (Ar-<u>C</u>H$_2$).

Mass Spectra (ES) Calc. for $C_{17}H_{12}O_{10}$: 376.04 g/mol. $[M-H]^-$. Found: 375.0351.

Tetraethyl-5,5'-methylenebis(2-hydroxyisophthalate) (**15**).

14 (26.34 g, 7 x 10^{-2} mol) and 98% sulfuric acid (33.12 g, 0.338 mol) were refluxed in ethanol (400 ml) for 24 hours. The reaction mixture was cooled before the flask was placed in an ice bath until a fluffy precipitate was formed. The precipitate was filtered and dried under suction. The filtrate was returned to the flask and the reaction was continued for 24 hours. The mixture was cooled, and the precipitate collected in the same way (30.62 g, 90 %).
Melting point = 83 – 85 °C (lit.[2] 84 – 85 °C)
^{1}H NMR (400 MHz, $CDCl_3$, δ, ppm): 11.73 (s, 2H, Ar-O$\underline{H}$), 7.83 (s, 4H, Ar$\underline{H}$), 4.40 (q, J = 7.13, 8H, COO-C$\underline{H}_2$), 3.90 (s, 2H, Ar-C$\underline{H}_2$), 1.40 (t, J = 7.11 Hz, 12H, CH_2-C$\underline{H}_3$).
^{13}C NMR (100 MHz, $CDCl_3$, δ, ppm): 167.73 ($\underline{C}OOC_2H_5$), 160.34 (Ar$\underline{C}$-$COOC_2H_5$), 136.19 (Ar$\underline{C}$H), 130.36 (Ar$\underline{C}$-CH_2), 117.20 (Ar$\underline{C}$-OH), 61.76 ($\underline{C}H_2$-CH_3), 39.58 (Ar-$\underline{C}H_2$), 14.36 (CH_2-$\underline{C}H_3$).
Mass Spectra (ES) Calc. for $C_{25}H_{28}O_{10}$: 488.468 g/mol. [M+ K^+]. Found: 527.1337.

Octaethyl-4,10,14,20-tetraoxa-1,3,11,13(1,4)-tetrabenzenacycloicosaphan-1^2,1^6,3^3,3^5,11^2,11^6,13^3,13^5-octacarboxylate (**2**).
Potassium carbonate (3.80 g, 2.75 x 10^{-2} mol) was added to dimethyl formamide (100 ml) in a twin necked round bottom flask and heated to 70°C. Into which a solution of **15** (1.34 g, 2.75 x 10^{-3} mol) and **16**[3] (2.16 g, 2.75 x 10^{-3} mol) in dimethylformamide (400 ml) was added gradually using a long-tipped dropping funnel which was submerged beneath the volume of solvent in the flask, over a period of 8 hours. The reaction was continued for 60 hours before filtering hot through a celite plug. The filtrate was added to saturated brine solution (800 ml) and extracted using 5 portions of ethyl acetate (200 ml). The organic layers were pooled, dried using magnesium sulfate and filtered, then concentrated under reduced pressure to yield the crude product. The crude residue was purified using column chromatography (n-hexane: ethyl acetate, 2:1) and yielded a white solid (0.86 g, 28 %).
Melting point = 164 – 165 °C
^{1}H NMR (400 MHz, DMSO-d_6, δ, ppm): 7.63 (s, 8H, Ar$\underline{H}$), 4.17 (q, J = 7.38 Hz, 16H, COO-C$\underline{H}_2$), 4.01 (s, 4H, Ar-C$\underline{H}_2$), 3.83 (t, J = 6.74 Hz, 8H, O-C$\underline{H}_2$), 1.61 (p, J = 6.92 Hz, 8H, O-CH_2-C$\underline{H}_2$), 1.30 (p, J = 7.40 Hz, 4H, O-CH_2-CH_2-C$\underline{H}_2$), 1.16 (t, J = 7.10 Hz, 24 H, COO-CH_2-C$\underline{H}_3$).
^{13}C NMR (100 MHz, DMSO-d_6, δ, ppm): 165.35 ($\underline{C}OOC_2H_5$), 155.24 (Ar$\underline{C}$-$COOC_2H_5$), 135.52 (Ar$\underline{C}$H), 133.53 (Ar$\underline{C}$-CH_2), 126.42 (Ar$\underline{C}$-O), 75.74 (O-$\underline{C}H_2$-C_2H_4), 60.94 (O-$\underline{C}H_2$-CH_3), 57.11 (Ar-$\underline{C}H_2$), 28.96 (O-CH_2-$\underline{C}H_2$), 21.02 (O-C_2H_4-$\underline{C}H_2$), 13.80 (O-CH_2-$\underline{C}H_3$).
Mass Spectra (ES) Calc. for $C_{60}H_{72}O_{20}$: 1112.46 g/mol. $[M+H]^+$. Found: 1113.4697.

4,10,14,20-Tetraoxa-1,3,11,13(1,4)-tetrabenzenacycloicosaphan-1^2,1^6,3^3,3^5,11^2,11^6,13^3,13^5-octacarboxylic acid (**1**).
2 (3.44 g, 3.09 x 10^{-3} mol) was dissolved in ethanol (300 ml) and added together with a solution of lithium hydroxide (3.00 g, 0.125 mol) in water (30 ml). The reaction was heated to reflux for 24 hours before it was cooled and concentrated under reduced pressure. Water (150 ml) was added and insoluble solids were removed using gravity filtration. Hydrochloric acid solution (5 M) was added until the filtrate turned milky. The suspended solid was removed by centrifuge, then dried yielding a white solid (2.60 g, 95 %).
Melting point = >300 °C
^{1}H NMR (400 MHz, DMSO-d_6, δ, ppm): 7.60 (s, 8H, Ar$\underline{H}$), 3.93 (s, 4H, Ar-C$\underline{H}_2$), 3.85 (t, J = 7.05 Hz, 8H, O-C$\underline{H}_2$), 1.60 (p, J = 7.15 Hz, 8H, OCH_2-C$\underline{H}_2$), 1.30 (p, J = 7.36 Hz, 4H, OC_2H_4-C$\underline{H}_2$).
^{13}C NMR (100 MHz, DMSO-d_6, δ, ppm): 167.11 ($\underline{C}OOH$), 155.06 (Ar$\underline{C}$-COOH), 135.57 (Ar$\underline{C}$H), 132.89 (Ar$\underline{C}$-CH_2), 127.48 (Ar$\underline{C}$-O), 75.75 (O-$\underline{C}H_2$-C_2H_4), 49.01 (Ar-$\underline{C}H_2$), 28.99 (OCH_2-$\underline{C}H_2$), 21.02 (OC_2H_4-$\underline{C}H_2$).
Mass Spectra (ES) Calc. for $C_{44}H_{40}O_{20}$: 888.111 g/mol. $[M+Na]^+$. Found: 911.2.

2,12-Dioxo-4,10,14,20-tetraoxa-1,3,11,13(1,4)-tetrabenzenacycloicosaphan-$1^2,1^6,3^3,3^5,11^2,11^6,13^3,13^5$-octacarboxylic acid (**4**).

1 (1.00 g, $1.13x10^{-3}$ mol) was dissolved in a solution of sodium hydroxide (1.00 g, 2.50×10^{-2} mol) in water (200 ml). Potassium permanganate (1.80 g, 1.13×10^{-2} mol) was added and the reaction stirred for 24 hours at 70 °C. Methanol (1 ml) was added to remove unreacted potassium permanganate and the reaction mixture filtered under suction. Hydrochloric acid solution (5 M) was added until the filtrate turned milky. The suspended solid was removed by centrifuge, then dried yielding a white solid (0.80 g, 77 %).

Melting point = >300 °C

^{1}H NMR (400 MHz, DMSO-d_6, δ, ppm): 8.11 (s, 8H, Ar$\underline{H}$), 4.08 (t, J = 7.01 Hz, 8H, O-$C\underline{H}_2$), 1.68 (p, J = 7.15 Hz, 8H, OCH_2-$C\underline{H}_2$), 1.42 (p, J = 7.04 Hz, 4H, OC_2H_4-$C\underline{H}_2$).

^{13}C NMR (100 MHz, DMSO-d_6, δ, ppm): 191.70 ($\underline{C}$O), 166.32 ($\underline{C}$OOH), 160.01 (Ar$\underline{C}$-COOH), 134.20 (Ar$\underline{C}$H), 130.91 (Ar$\underline{C}$-CH_2), 127.57 (Ar$\underline{C}$-O), 75.46 (O-$\underline{C}H_2$-C_2H_4), 28.08 (OCH_2-$\underline{C}H_2$), 20.48 (OC_2H_4-$\underline{C}H_2$).

Mass Spectra (ES) Calc. for $C_{44}H_{36}O_{22}$: 916.17 g/mol. $[M-H]^-$. Found: 915.162.

2,12-Dihydroxy-4,10,14,20-tetraoxa-1,3,11,13(1,4)-tetrabenzenacycloicosaphan-$1^2,1^6,3^3,3^5,11^2,11^6,13^3,13^5$-octacarboxylic acid (**3**).

4 (0.80 g, 8.73×10^{-4} mol) was treated with a solution of sodium borohydride (0.66 g, 1.75×10^{-2} mol) in water (100 ml), and the reaction was stirred at room temperature for 24 hours. Following this the reaction mixture was cooled in ice and hydrochloric acid (5 M) was added to remove unreacted sodium borohydride. The reaction mixture was warmed to room temperature and 37 % hydrochloric acid was added until it became milky. The suspended solids were removed by centrifuge, then dried yielding a white solid (0.80 g, 100 %).

Melting point = >300 °C

^{1}H NMR (400 MHz, NaOD/D_2O, δ, ppm): 7.31 (s, 8H, Ar$\underline{H}$), 5.76 (s, 2H, Ar-C$\underline{H}$-OH), 3.89 (t, J = 7.94 Hz, 8H, O-$C\underline{H}_2$), 1.52 (p, J = 7.30 Hz, 8H, O-CH_2-$C\underline{H}_2$), 1.22 (p, J = 7.30 Hz, 4H, O-C_2H_4-$C\underline{H}_2$).

^{13}C NMR (100 MHz, DMSO-d_6, δ, ppm): 167.06 ($\underline{C}$OOH), 155.81 (Ar$\underline{C}$-COOH), 133.98 (Ar$\underline{C}$H), 130.11 (Ar$\underline{C}$-CH_2), 127.02 (Ar$\underline{C}$-O), 77.22 ($\underline{C}$-OH), 75.88 (O-$\underline{C}H_2$-C_2H_4), 29.02 (O-CH_2-$\underline{C}H_2$), 21.04 (O-C_2H_4-$\underline{C}H_2$).

Mass Spectra (ES) Calc. for $C_{44}H_{40}O_{22}$: 920.201 g/mol. $[M- H]^-$. Found: 919.1934

Reoxidation of **3** into **4**.

The $KMnO_4$ oxidation procedure which converted **1** to **4** was applied to **3** at the same scale. The white solid obtained (85%) was shown to be entirely **4** by ^{1}H NMR analysis.

Supplementary note 1. Spectral characterization for **1**, **4** and **3**.

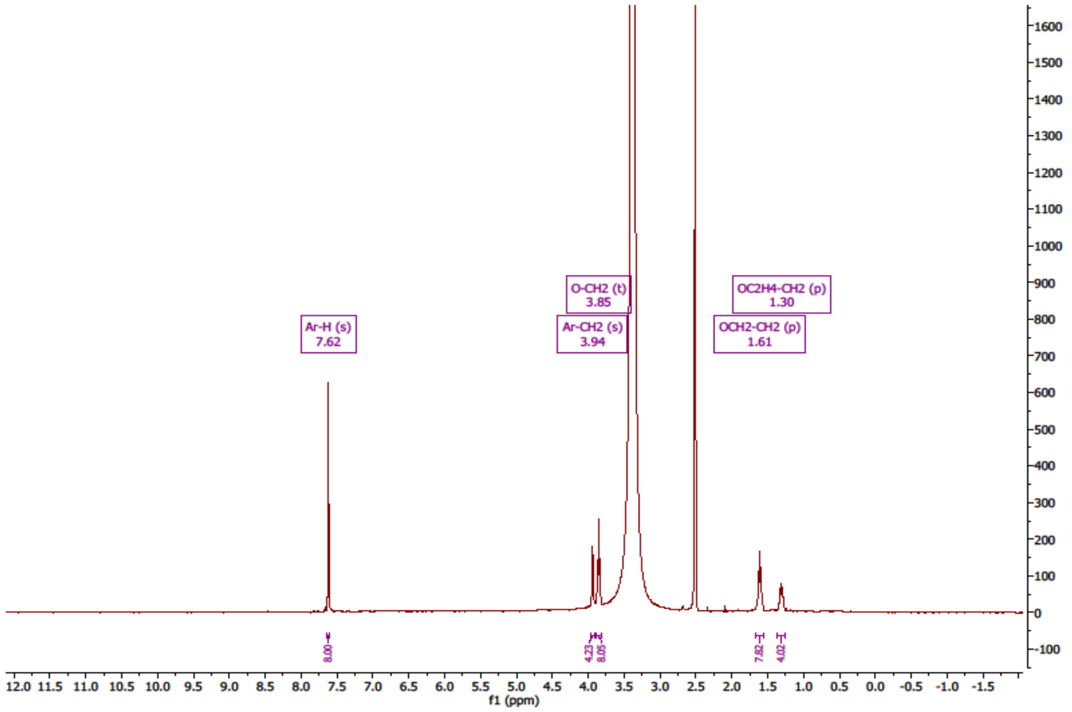

Supplementary Fig. 2a. ^{1}H NMR spectrum (400 MHz, DMSO-d_6) of **1**.

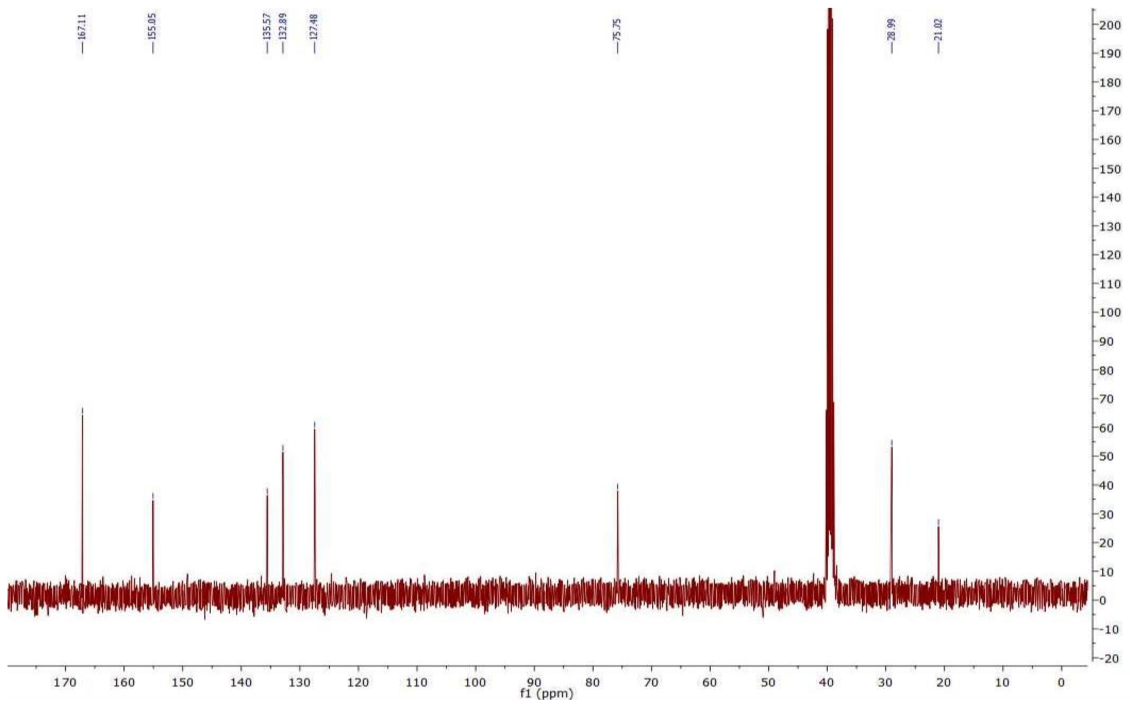

Supplementary Fig. 2b. ^{13}C NMR spectrum (100 MHz, DMSO-d_6) of **1**.

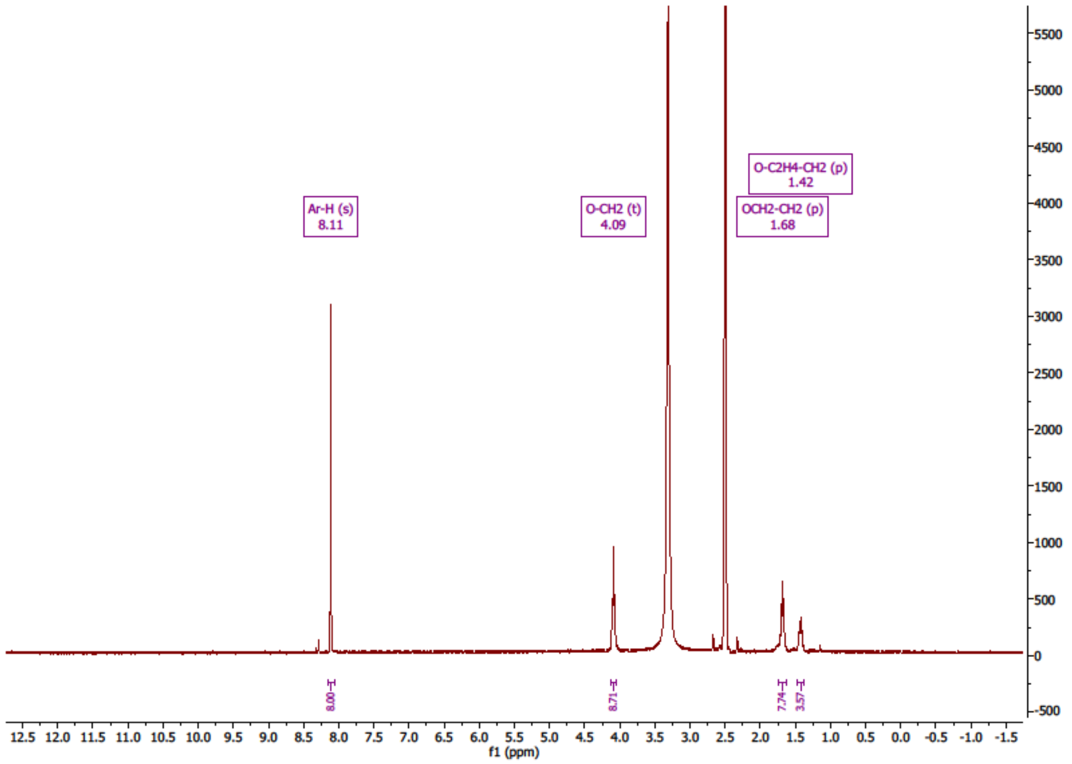

Supplementary Fig. 2c. ^{1}H NMR spectrum (400 MHz, DMSO-d_6) of **4**.

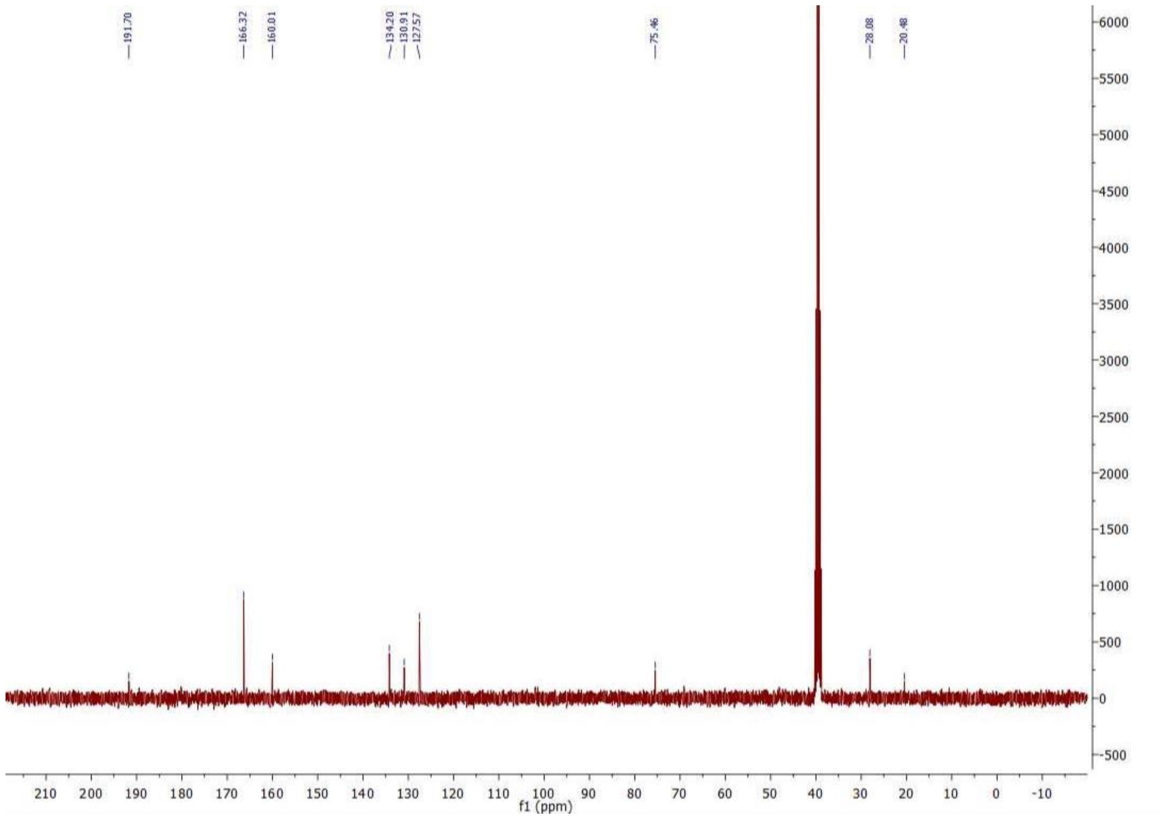

Supplementary Fig. 2d. ^{13}C NMR spectrum (100 MHz, DMSO-d_6) of **4**.

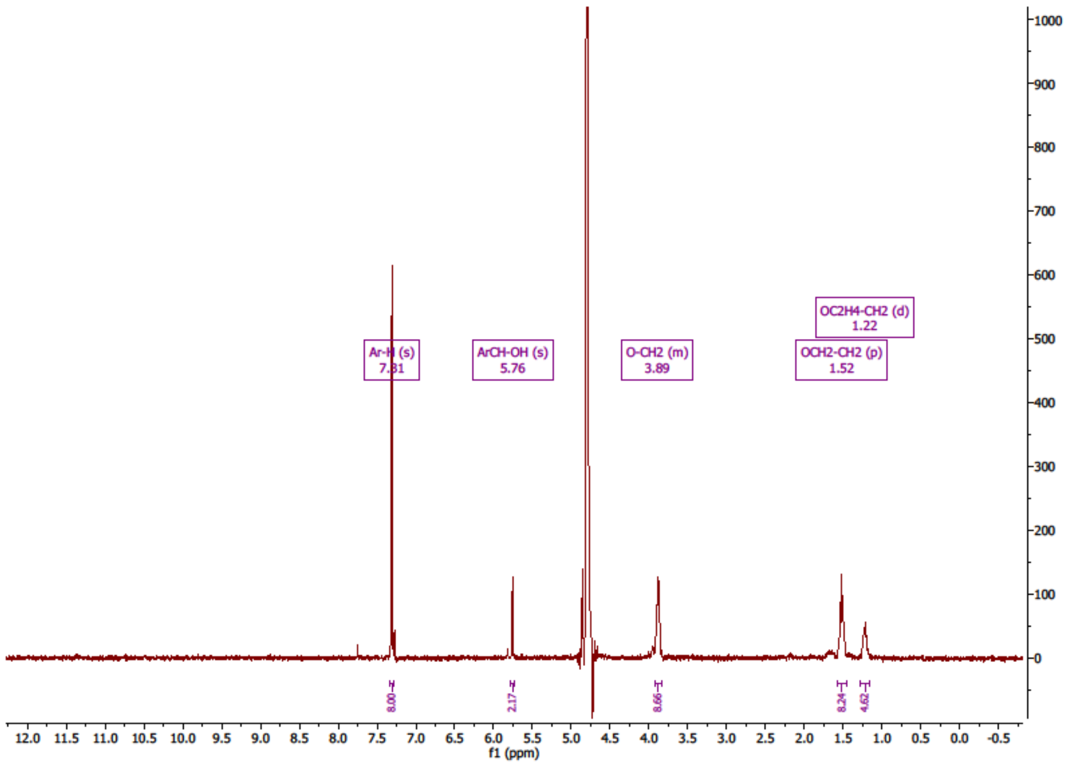

Supplementary Fig. 2e. ^{1}H NMR spectrum (400 MHz, DMSO-d_6) of **3**.

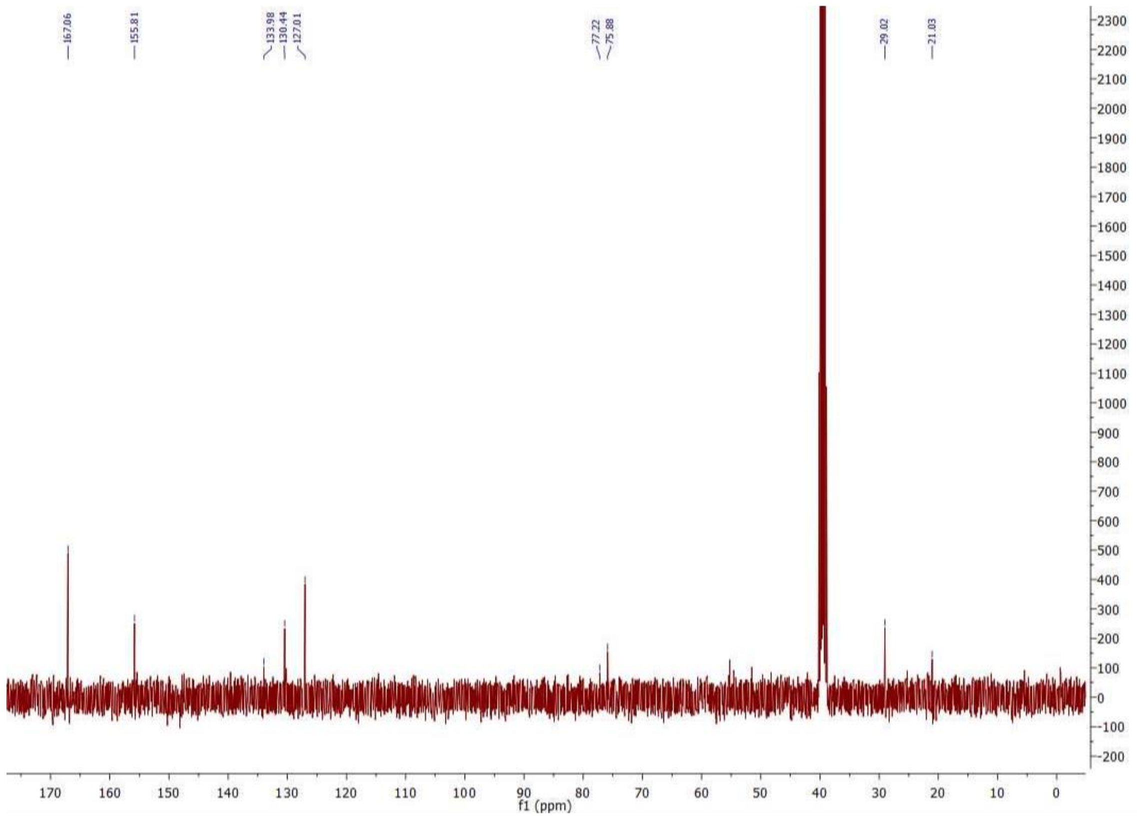

Supplementary Fig. 2f. ^{13}C NMR spectrum (100 MHz, DMSO-d_6) of **3**.

Supplementary methods 2. Synthesis scheme, preparative procedures and characterizations of the ancillary cyclophanes and other compounds.

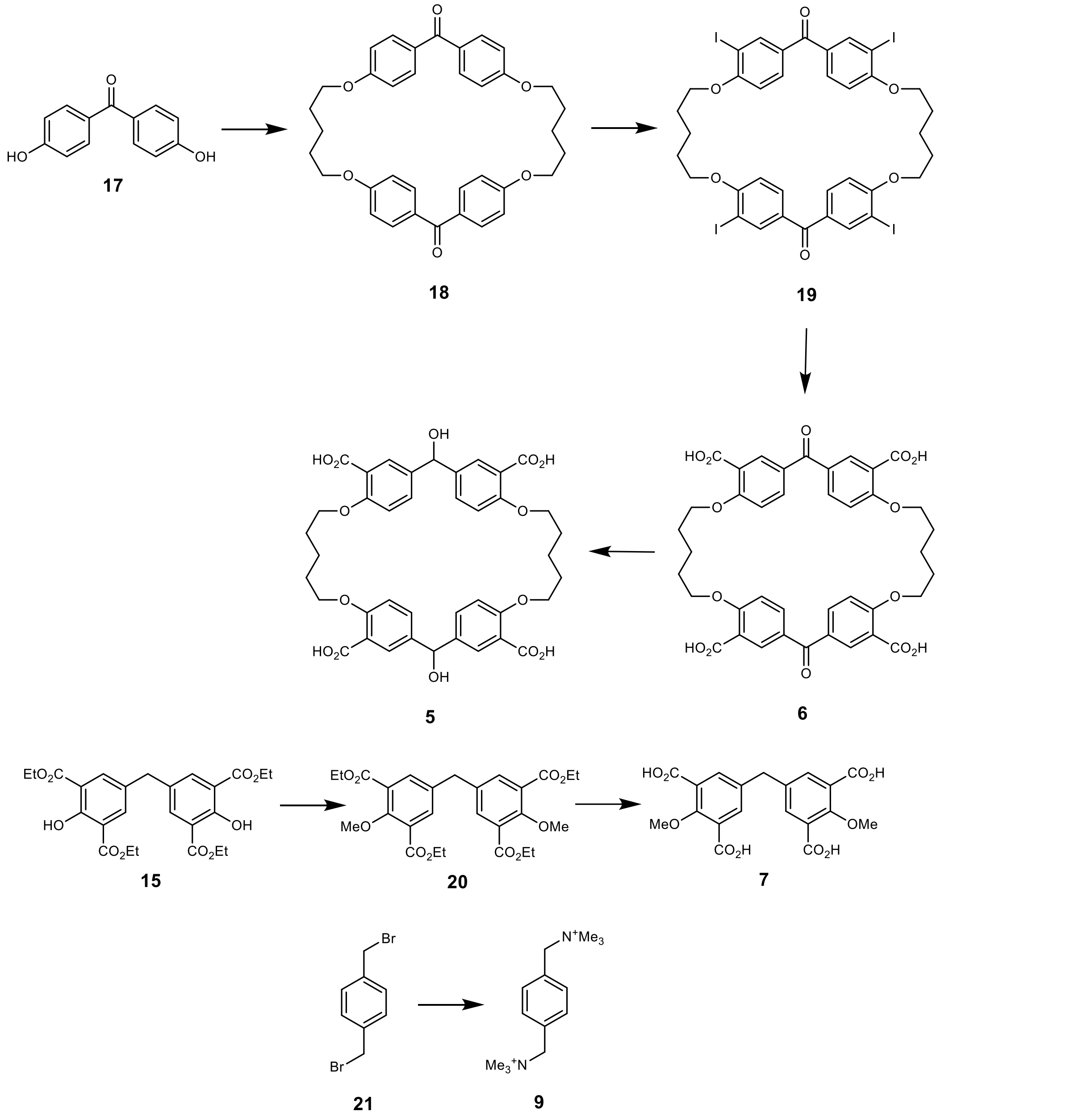

Supplementary Fig. 3. Scheme for the synthesis of cyclophanes **5** and **6**, and compounds **7** and **9**.

Preparative Procedures

2,12-Dioxo-4,10,14,20-tetraoxa-1,3,11,13(1,4)-tetrabenzenacycloicosaphane (**18**)[4].

4,4'-Dihydroxybenzophenone **17** (3.20 g, 14.9 mmol) and diiodopentane (4.86 g, 15.0 mmol) were dissolved in acetone (400 ml, freshly distilled HPLC grade, dried over anhydrous potassium carbonate) and slowly added, with the aid of a dropping funnel whose capillary tip is dipping below the surface, to a refluxing suspension of acetone (600 ml) and cesium carbonate (8.0 g, 25 mmol) over 12 hours. The colourless solution turned a pale yellow colour. This mixture was then refluxed for a further three days. The hot reaction mixture was then filtered through a silica/hyflo plug and the filtrate chilled to 4 °C whereupon a white solid precipitated out. The solid was purified using flash silica chromatography eluting with dichloromethane: ethyl acetate (95:5 v/v) yielding the desired white plate-like solid (0.8 g, 10%).
Melting Point = 187-188 °C.
Elemental analysis; Found: C, 74.46; H, 6.02. Required for $C_{36}H_{36}O_6.H_2O$: C, 74.22; H, 6.53.
^{1}H NMR ($CDCl_3$, 500 MHz, δ, ppm): 7.68(d, 8H, Ar$\underline{H}$, J=8.7Hz), 6.83(d, 8H, Ar$\underline{H}$, J=8.7Hz), 4.10(t, 8H, O-C$\underline{H}_2$, J=6.0 Hz), 1.87(m, 8H, O-CH_2-C$\underline{H}_2$, J=6.0, 6.6 Hz), 1.72(t, 4H, O-$(CH_2)_2$-C$\underline{H}_2$, J=6.6 Hz).
^{13}C NMR ($CDCl_3$, 100 MHz, δ, ppm): 194.31, 162.03, 132.08, 130.64, 114.12, 67.53, 27.63, 21.72.
Mass Spectra (EI): 564(61, M^+), 552(4), 509(3), 368(4), 283(9), 227(6), 215(7), 197(5), 185(6), 157(4), 139(4), 121(53), 83(100), 69(35), 59(24).

2,12-Dioxo-1^2,3^3,11^2,13^3-tetraiodo-4,10,14,20-tetraoxa-1,3,11,13(1,4)-tetrabenzenacycloicosaphane (**19**)[4].
18 (0.30 g, 0.53 mmol) was dissolved in hot chloroform (25 ml, 0.31 mol), dried over anhydrous potassium carbonate) and to this solution was added silver trifluoroacetate (1.00 g, 4.9 mmol) and iodine (1.24 g, 4.9 mmol). The reaction vessel was sealed with a stopper and stirred vigorously for four days. The resultant mixture was then refluxed with chloroform (400 ml) for one hour and filtered hot through a hyflo supercel plug and the filtrate was collected. The yellow silver residue present on the hyflo was washed several times with hot chloroform. The combined filtrate was evaporated to dryness to give a black solid. The solid was then treated with hot ethanol (200 ml) and refluxed for one hour, cooled and filtered. The resultant off-white solid was then washed with cold ethanol and dried (0.35 g, 92%).
Melting Point = 282-283 °C.
Elemental analysis; Found: C, 38.34; H, 2.81. Required for $C_{36}H_{32}O_6I_4.3H_2O$: C, 38.50; H, 3.38.
^{1}H NMR ($CDCl_3$, 500 MHz, δ, ppm): 8.10(s, 4H, Ar$\underline{H}$), 7.69(d, 4H, Ar$\underline{H}$, J=8.7 Hz), 6.79(d, 4H, Ar$\underline{H}$, J=8.6 Hz), 4.23(t, 8H, O-C$\underline{H}_2$, J=5.6 Hz), 1.98(m, 8H, O-CH_2-C$\underline{H}_2$, J=6.0, 6.6 Hz), 1.85(t, 4H, O-$(CH_2)_2$-C$\underline{H}_2$, J=6.6 Hz).
^{13}C NMR (DMSO, 100 MHz, δ, ppm) 178.64, 160.85, 140.85, 132.04, 131.79, 112.76, 87.06, 69.80, 28.13, 22.43
Mass Spectra (EI): 1068(26, M^+), 941(9), 815(3), 591(9), 564(44), 368(32), 280(26), 246(31), 203(100).

2,12-Dioxo-4,10,14,20-tetraoxa-1,3,11,13(1,4)-tetrabenzenacycloicosaphan-1^2,3^3,11^2,13^3-tetracarboxylic acid (**6**)[4].
19 (300 mg, 0.0280 mmol) and tetrakis(triphenylphosphine)-Palladium-(0) (150 mg, 0.130 mmol) were suspended in dimethylformamide (45 ml) in a three necked round bottomed flask (100 ml). Potassium carbonate (3.0 g, 22 mmol) dissolved in water (6 ml) was added and stirred. A carbon monoxide balloon was attached via a tap outlet. The reaction vessel was

degassed using another tap outlet and a water pump. Once the system was fully degassed, carbon monoxide was passed through the system until saturation. At this point the balloon tap was opened and the reaction mixture stirred at 60 °C overnight. The mixture was cooled, water (90 ml) added and the aqueous layer washed with dichloromethane (3x30 ml). The aqueous layer was then acidified using dilute hydrochloric acid. This produced a brown solid, which was filtered and dried (0.11 g, 48%).
Melting Point = >300 °C.
Elemental analysis; Found: C, 62.82; H, 5.00. Required for $C_{40}H_{36}O_{14}.H_2O$: C, 63.32; H, 5.01.
^{1}H NMR (D_2O/NaOD, 300MHz, δ, ppm): 7.58(s, 4H, Ar<u>H</u>), 7.18(d, 4H, Ar<u>H</u>, J=8.8 Hz), 6.69(d, 4H, Ar<u>H</u>, J=8.8 Hz), 4.02(t, 8H, O-C<u>H</u>$_2$, J=5.6 Hz), 1.72(m, 8H, O-CH$_2$-C<u>H</u>$_2$, J=5.6, 5.4 Hz), 1.52(t, 4H, O-(CH$_2$)$_2$-C<u>H</u>$_2$, J=5.4 Hz).
^{13}C NMR (DMSO, 100 MHz, δ, ppm) 192.32, 167.04, 160.78, 135.07, 132.83, 129.06, 122.14, 113.41, 68.76, 27.29, 21.50.
Mass Spectra (EI): 741(100, M + H$^+$), 724(8), 697(12), 651(4), 398(3), 317(8), 301(9).

2,12-Dihydroxy-4,10,14,20-tetraoxa-1,3,11,13(1,4)-tetrabenzenacycloicosaphan-1^2,3^3,11^2,13^3-tetracarboxylic acid (**5**)[4].
6 (0.070 g, 0.094 mmol) was dissolved in water (5.0 ml, 280 mmol) with the aid of a few drops of dilute sodium hydroxide and then sodium borohydride (0.10 g, 2.6 mmol) added and stirred overnight at room temperature (ca. 20 °C). A few drops of acetic acid were then added to destroy any unreacted sodium borohydride. The mixture was then acidified to approximately pH 1 with dilute hydrochloric acid. The precipitated product was then filtered, washed with water and dried (0.04 g, 57%).
Melting Point = >300 °C.
Elemental analysis; Found: C, 62.82; H, 5.00. Required for $C_{40}H_{40}O_{14}.H_2O$: C, 62.99; H, 5.51.
^{1}H NMR (D_2O/NaOD, 300MHz, δ, ppm): 7.45(s, 4H, Ar<u>H</u>), 7.03(d, 4H, Ar<u>H</u>, J=7.4 Hz), 6.92(d, 4H, Ar<u>H</u>, J=7.2 Hz), 5.77(d, 2H, -C<u>H</u>-OH), 4.06(b, 8H, O-C<u>H</u>$_2$), 1.74(b, 8H, O-CH$_2$-C<u>H</u>$_2$), 1.54(b, 4H, O-(CH$_2$)$_2$-C<u>H</u>$_2$).
^{13}C NMR (DMSO, 100 MHz, δ, ppm) 168.00, 156.58, 137.90, 130.68, 128.47, 113.71, 97.10, 70.53, 68.70, 28.26, 22.09.
Mass Spectra (EI): 744(100, M$^+$), 728(4), 701(17), 655(4), 402(3), 321(5), 305(9).

Tetraethyl 5,5'-methylenebis(2-methoxyisophthalate) (**20**)
K_2CO_3 (5.80 g) is added into a round bottomed flask. **15** (2.05 g, 4.2 mmol) is dissolved in dimethylformamide (200 ml) and the solution is then added into the flask. The oil bath is set at 60 °C. Iodomethane (1.1 ml) is added quickly and then the mixture is refluxed for 24 hours. dimethylformamide is removed by rotavapor. Water is added until all K_2CO_3 is dissolved. Ethyl acetate (100 ml) is used to extract the reaction mixture (four times). Then the solvent is removed by rotavapor to leave a white solid (3.9 g, 93%).
^{1}H NMR (CDCl$_3$, 300 MHz, δ, ppm); 7.70(s, 4H, Ar<u>H</u>), 4.73 (q, J=7.1 Hz, 8H, OC<u>H</u>$_2$CH$_3$), 3.96 (s, 2H, ArC<u>H</u>$_2$), 3.90 (s, 6H, 8, 30), 1.38 (t, J=7.1 Hz, 12H, OCH$_2$C<u>H</u>$_3$).

5,5'-Methylenebis(2-methoxyisophthalic acid) (**7**)[5].
LiOH (1.86 g) is dissolved in water (50 ml) and **20** (4.00 g. 7.7 mmol) is dissolved in methanol (200 ml). The mixture is poured into a round bottomed flask and heated to reflux for 20 hours. The solvent is removed by rotavapor and 4M HCl is added until a precipitate is seen. The precipitate is filtered under suction and dried in the oven to give a white solid (2.8 g, 89%).
^{1}H NMR (300 MHz, DMSO-d$_6$, δ, ppm): 7.70(s, 4H, Ar<u>H</u>), 4.01(s, 2H, ArC<u>H</u>$_2$), 3.76(s, 6H, OC<u>H</u>$_3$).

Trimethyl-4-[(1,1,1-trimethylammonio)methyl]benzyl ammonium dibromide (**9**)[6].
α,α’-Dibromo-*p*-xylene **21** (0.50 g, 1.9 mmol) was dissolved in dimethylformamide (50 ml). Trimethylamine (0.6 ml, 3.8 mmol) was then added slowly with stirring. After 10 min a white precipitate formed and the suspension was stirred for a further 20 hours. The precipitate was then filtered and dried yielding a white solid (0.71 g, 98%).
Melting point = 349-350 °C (lit.[6] >320 °C).
^{1}H NMR (D_2O, 500 MHz, δ, ppm): 7.46(s, 4H, Ar<u>H</u>), 4.32(s, 4H, ArC<u>H</u>$_2$), 2.88(s, 18H, N^+C<u>H</u>$_3$).
^{13}C NMR (DMSO, 100 MHz, δ, ppm) 133.66, 130.79, 67.57, 52.32.
Mass Spectra (EI): 407, 405, 403(6, M^++Na^+), 303, 301(100, M^+-Br^-), 284(10), 267(8), 244(12), 214(20).

Supplementary note 2. Photographs of aqueous solutions formed by various mixtures of **1** and **10**.

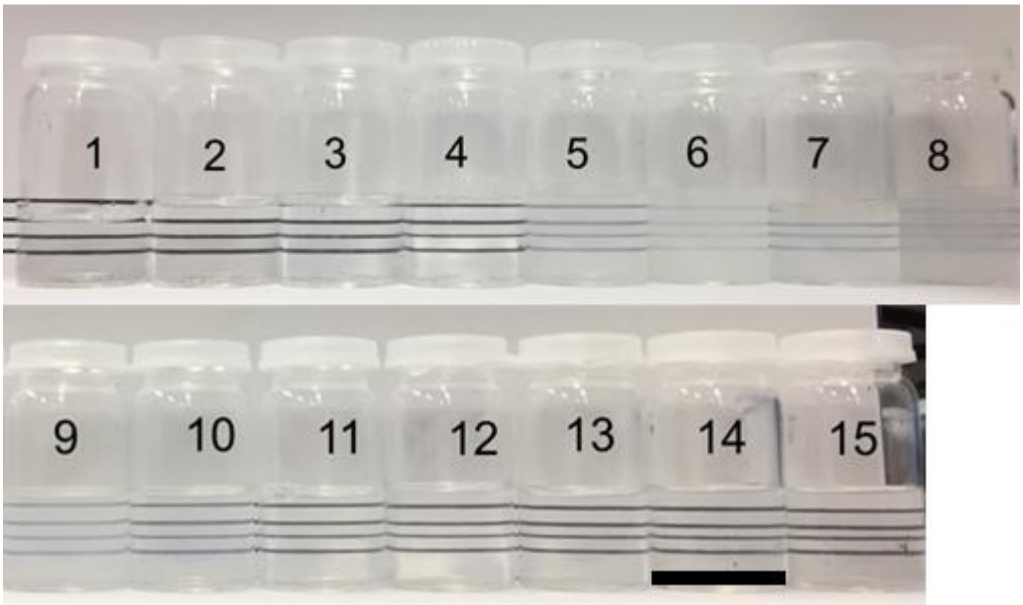

Supplementary Fig. 4. Photographs of aqueous solutions formed by mixtures of **1** and **10** with the following molar fractions of **10** (X_{10}): 1; 0.000, 2; 0.107, 3; 0.186, 4; 0.478, 5; 0.647, 6; 0.786, 7; 0.880, 8; 0.936, 9; 0.967, 10; 0.971, 11; 0.976, 12; 0.983, 13; 0.996, 14; 0.998, 15; 1.000. Scale bar is 2.0 cm.

Supplementary note 3. Dynamic light scattering (DLS) data.

Size Distribution Report by Intensity

v2.2

Sample Details

Sample Name: APsampleCH2 1

SOP Name: mansettings.nano

General Notes:

File Name:	20200130.dts	Dispersant Name:	Water
Record Number:	1	Dispersant RI:	1.330
Material RI:	1.59	Viscosity (cP):	0.8872
Material Absorbtion:	0.010	Measurement Date and Time:	30 Jan 2020 13:34:00

System

Temperature (°C):	24.9	Duration Used (s):	70
Count Rate (kcps):	188.0	Measurement Position (mm):	4.65
Cell Description:	Glass cuvette with square a...	Attenuator:	5

Results

			Size (d.nm)	% Intensity:	St Dev (d.nm)
Z-Average (d.nm):	163.4	Peak 1:	178.5	100.0	54.75
PdI:	0.077	Peak 2:	0.000	0.0	0.000
Intercept:	0.964	Peak 3:	0.000	0.0	0.000
Result	Good				

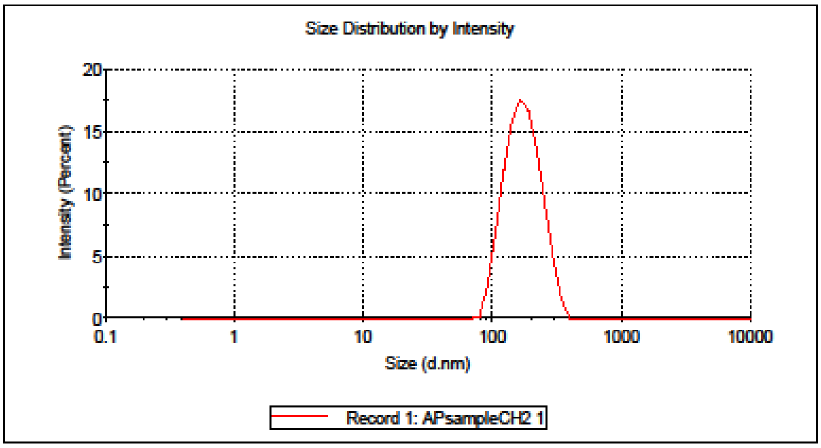

Supplementary Fig. 5a. DLS data of **1** and **10** (1:8) in aqueous solution, pH 10 in all cases.

Size Distribution Report by Intensity

v2.2

Sample Details

Sample Name:	AP sample CO 1
SOP Name:	mansettings.nano
General Notes:	

File Name:	20191202.dts	Dispersant Name:	Water
Record Number:	19	Dispersant RI:	1.330
Material RI:	1.59	Viscosity (cP):	0.8872
Material Absorbtion:	0.010	Measurement Date and Time:	23 Dec 2019 16:40:53

System

Temperature (°C):	24.9	Duration Used (s):	60
Count Rate (kcps):	237.3	Measurement Position (mm):	4.65
Cell Description:	Glass cuvette with square a...	Attenuator:	6

Results

			Size (d.nm)	% Intensity:	St Dev (d.nm)
Z-Average (d.nm):	175.7	Peak 1:	194.0	100.0	65.64
PdI:	0.118	Peak 2:	0.000	0.0	0.000
Intercept:	0.957	Peak 3:	0.000	0.0	0.000
Result	Good				

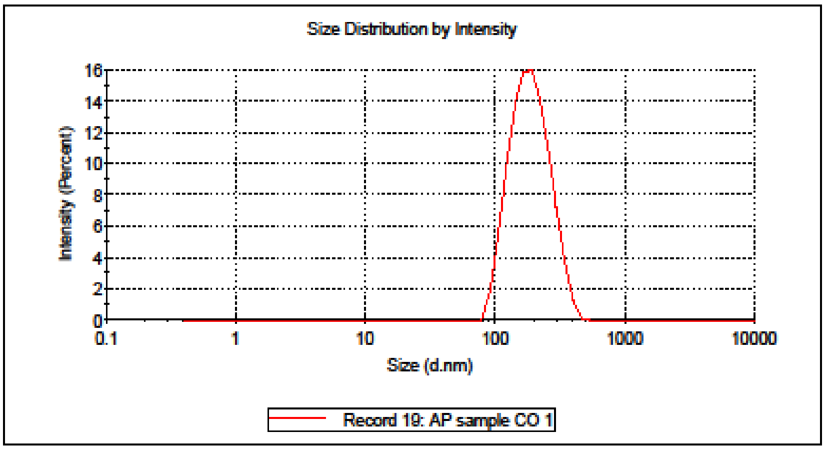

Malvern Instruments Ltd
www.malvern.com

Zetasizer Ver. 6.20
Serial Number : MAL1051366

File name: 20191202-26
Record Number: 19
18 2 2025 17:24:38

Supplementary Fig. 5b. DLS data of **4** and **10** (1:8) mixture.

Size Distribution Report by Intensity

v2.2

Sample Details

Sample Name: APsampleOH 1

SOP Name: mansettings.nano

General Notes:

**File Name:**	20200130.dts	**Dispersant Name:**	Water
**Record Number:**	4	**Dispersant RI:**	1.330
**Material RI:**	1.59	**Viscosity (cP):**	0.8872
**Material Absorbtion:**	0.010	**Measurement Date and Time:**	30 Jan 2020 14:37:58

System

**Temperature (°C):**	25.1	**Duration Used (s):**	60
**Count Rate (kcps):**	281.9	**Measurement Position (mm):**	1.05
**Cell Description:**	Glass cuvette with square a...	**Attenuator:**	4

Results

			Size (d.nm)	% Intensity:	St Dev (d.nm)
**Z-Average (d.nm):**	447.5	**Peak 1:**	614.6	93.7	462.4
**PdI:**	0.350	**Peak 2:**	4158	6.3	991.1
**Intercept:**	0.653	**Peak 3:**	0.000	0.0	0.000

Result Good

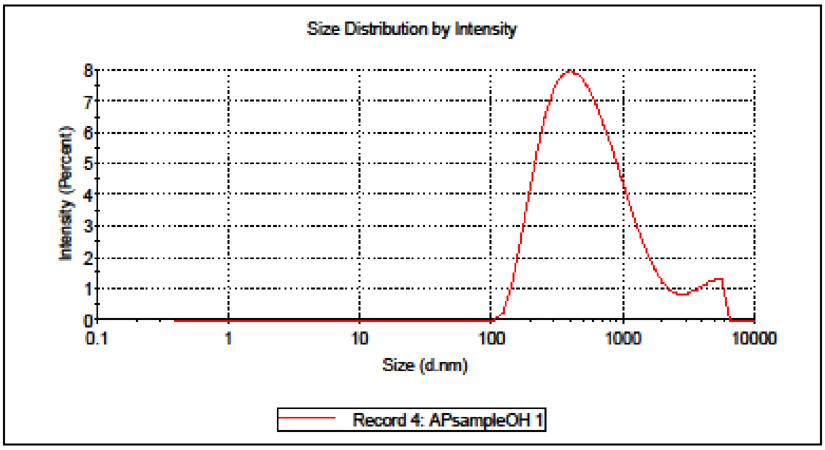

Supplementary Fig. 5c. DLS data of **3** and **10** (1:8) mixture.

Supplementary note 4. Nanoparticle tracking analysis (NTA) data.

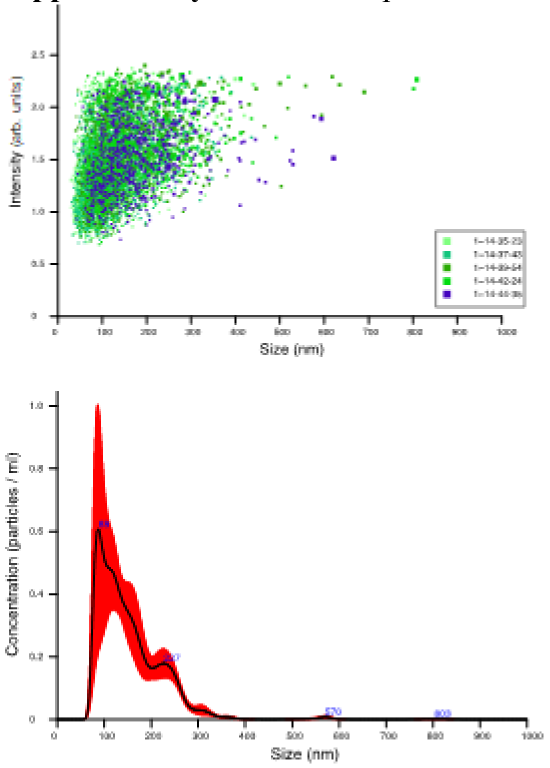

Supplementary Fig. 6a. NTA data of **1** and **10** (1:8) mixture in water, pH 10 in all cases.

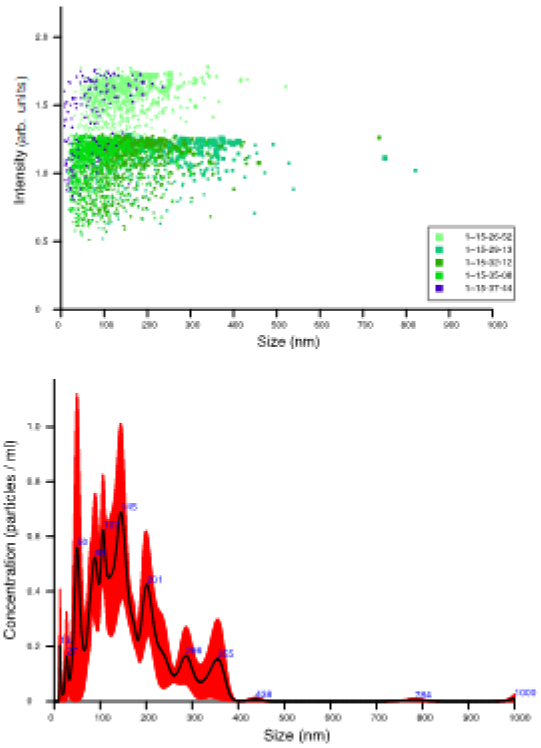

Supplementary Fig. 6b. NTA data of **4** and **10** (1:8) mixture.

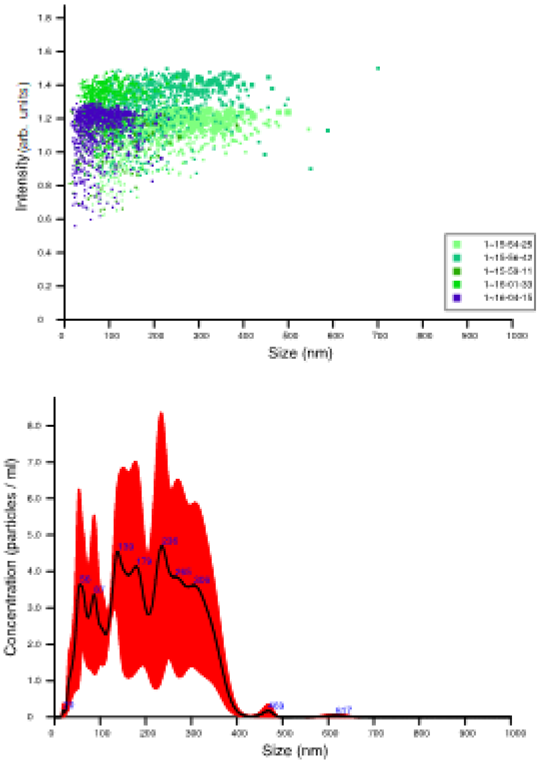

Supplementary Fig. 6c. NTA data of **3** and **10** (1:8) mixture.

Supplementary note 5. Aggregation of detergents with/without cyclophanes.

Supplementary Table 1. Cmc or cac values of detergents with/without cyclophanes in water.

	cmc or cac[a] (10^{-3} M)
**11**	6.5 (8.3[b])
**11** +**1**	4.9
**11** +**4**	4.9
**11** +**3**	4.9
**12**	0.30 (0.30[b])
**12** +**1**	0.18
**12** +**4**	0.36
**12** +**3**	0.36
**10**	1.0 (1.0[c])(1.4[b])
**10** +**1**	0.15 (0.09[c])
**10** +**4**	0.16 (0.22[c])
**10** +**3**	0.18 (0.22[c])

a. critical micelle concentration or critical aggregation concentration. Determined via fluorescence of hydrophobic probe 9,10-diphenylanthracene (λ_{ex} 383, λ_{em} 414 nm).
b. literature value[4,5].
c. determined by the pendant drop method[6,7].

Supplementary note 6. Transmission electron microscopy (TEM) images (enlarged).

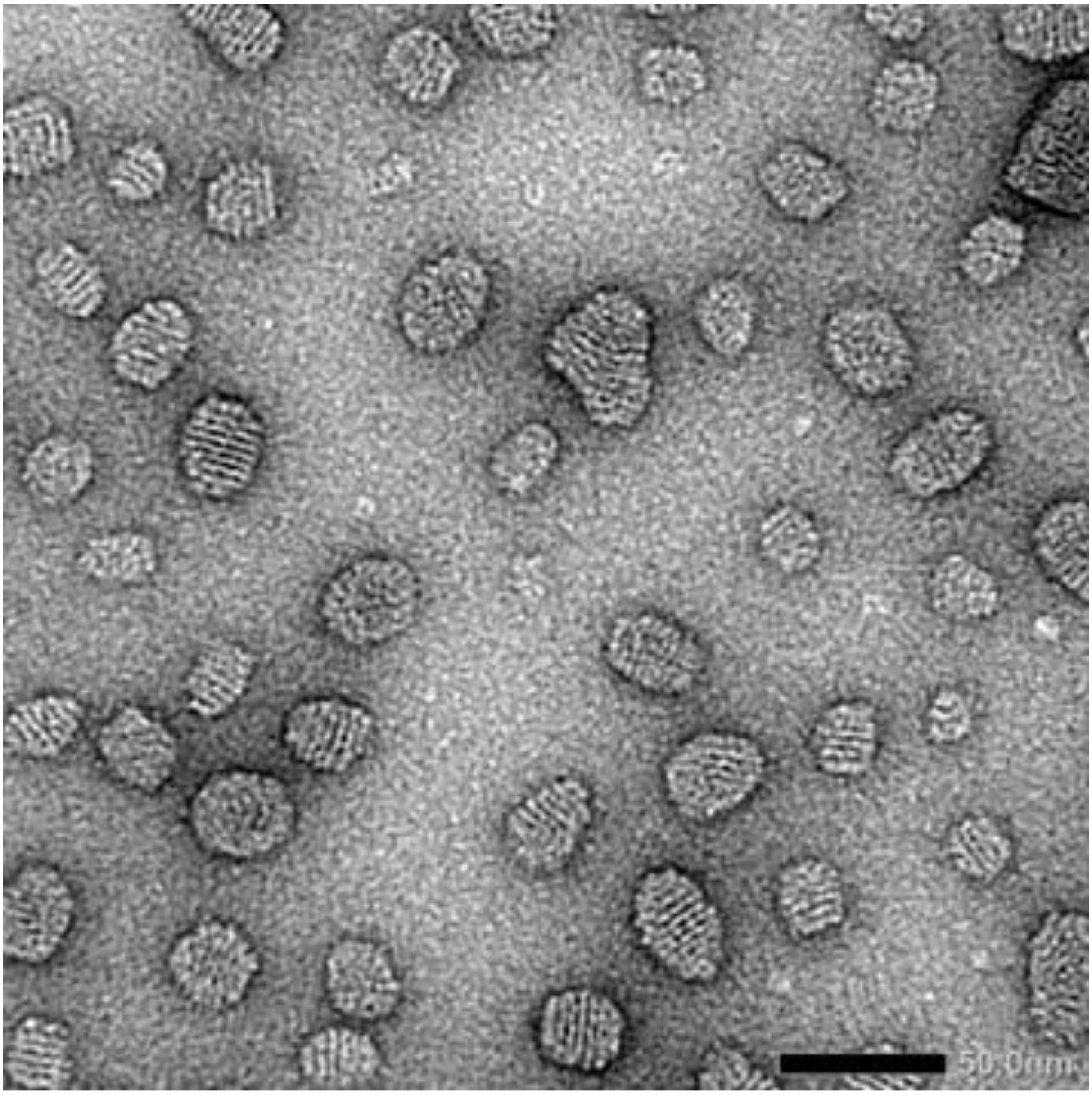

Supplementary Fig. 7a. TEM images of **1** and **10** (1:8) mixture. Scale bar label is 50.0 nm.

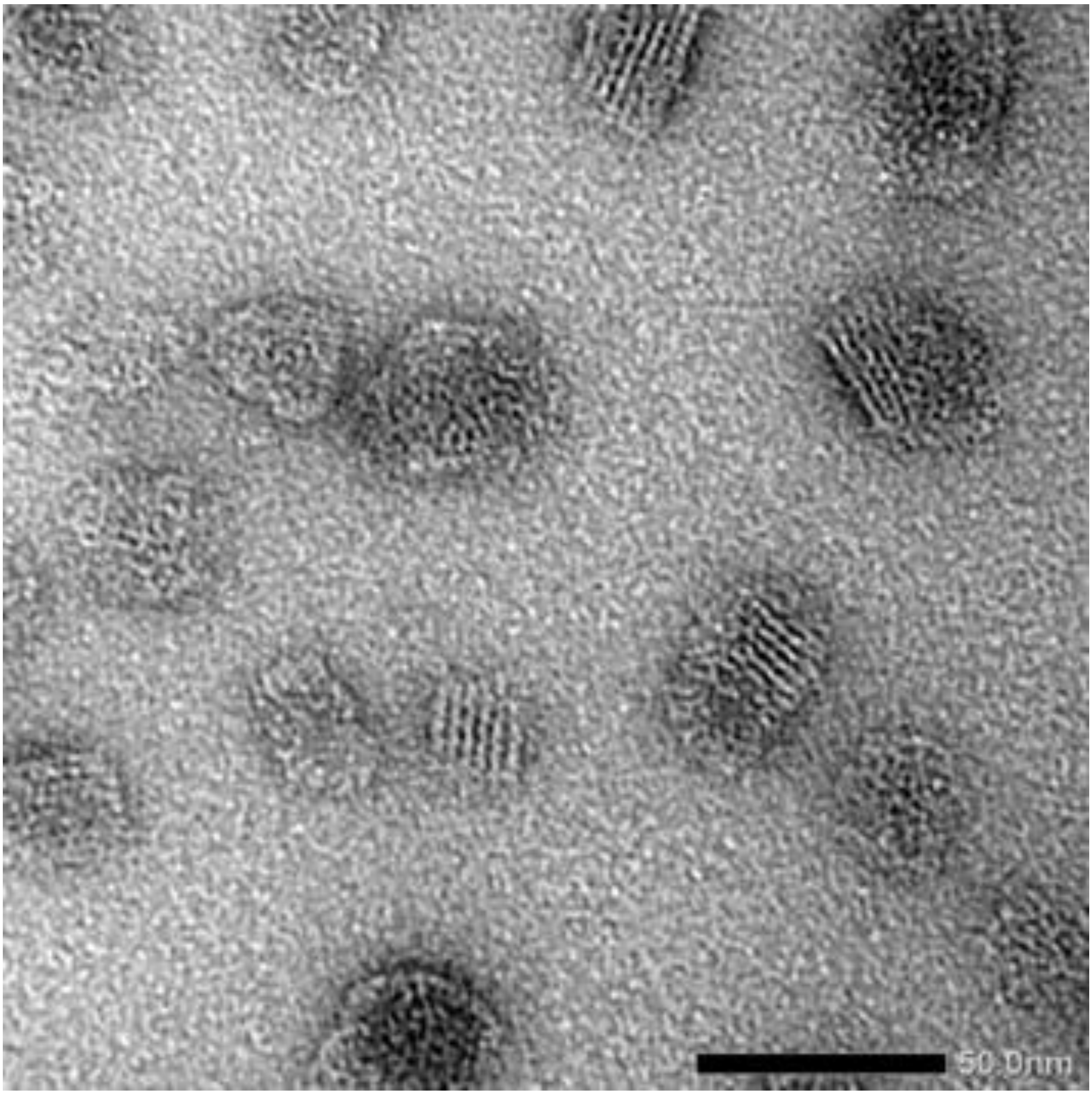

Supplementary Fig. 7b. TEM images of **3** and **10** (1:8) mixture. Scale bar label is 50.0 nm.

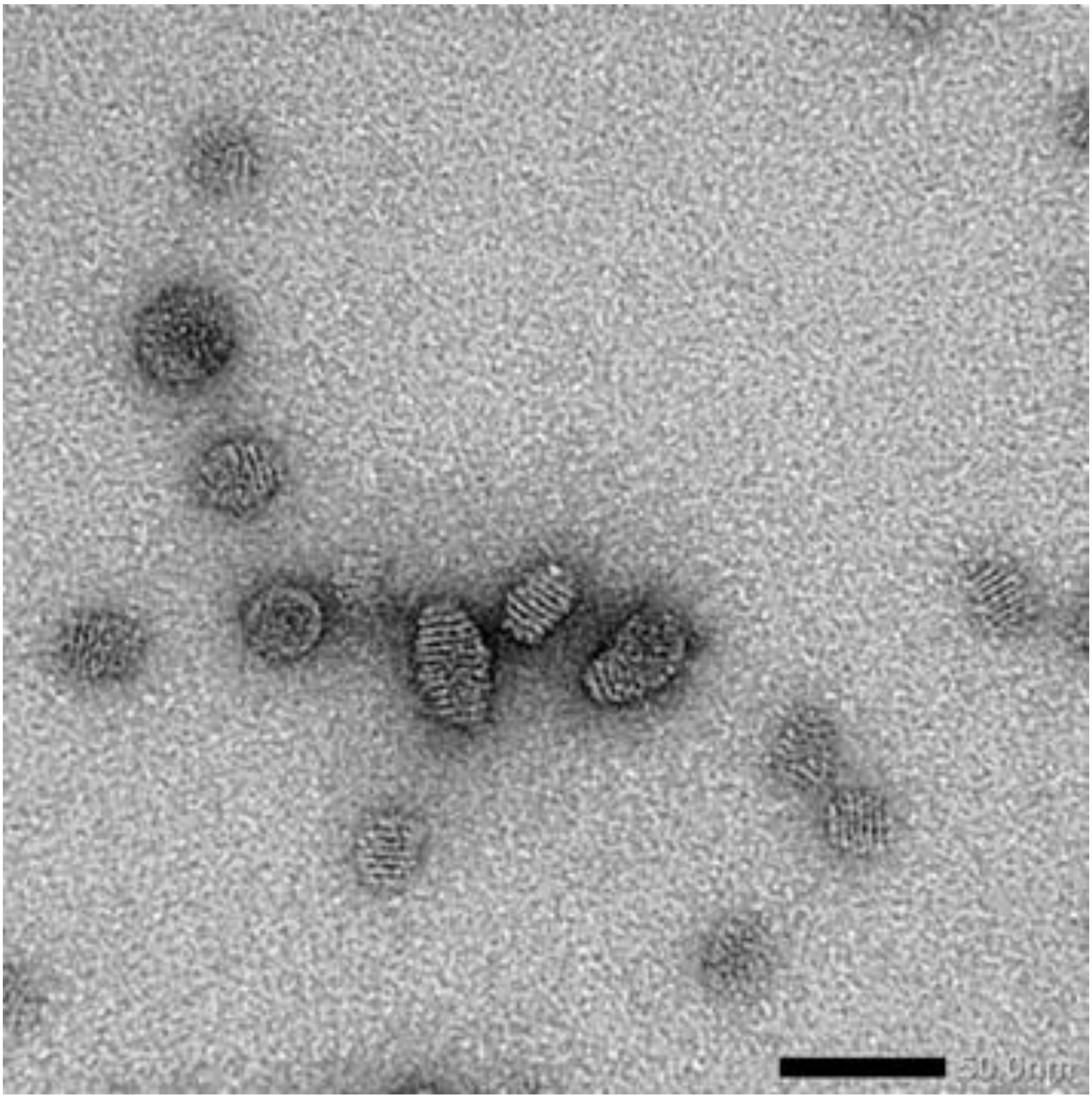

Supplementary Fig. 7c. TEM images of **4** and **10** (1:8) mixture. Scale bar label is 50.0 nm.

Supplementary note 7. ^{1}H NMR spectral testing of the binding abilities of hosts **4**, **3** and **1** with guest **9**.

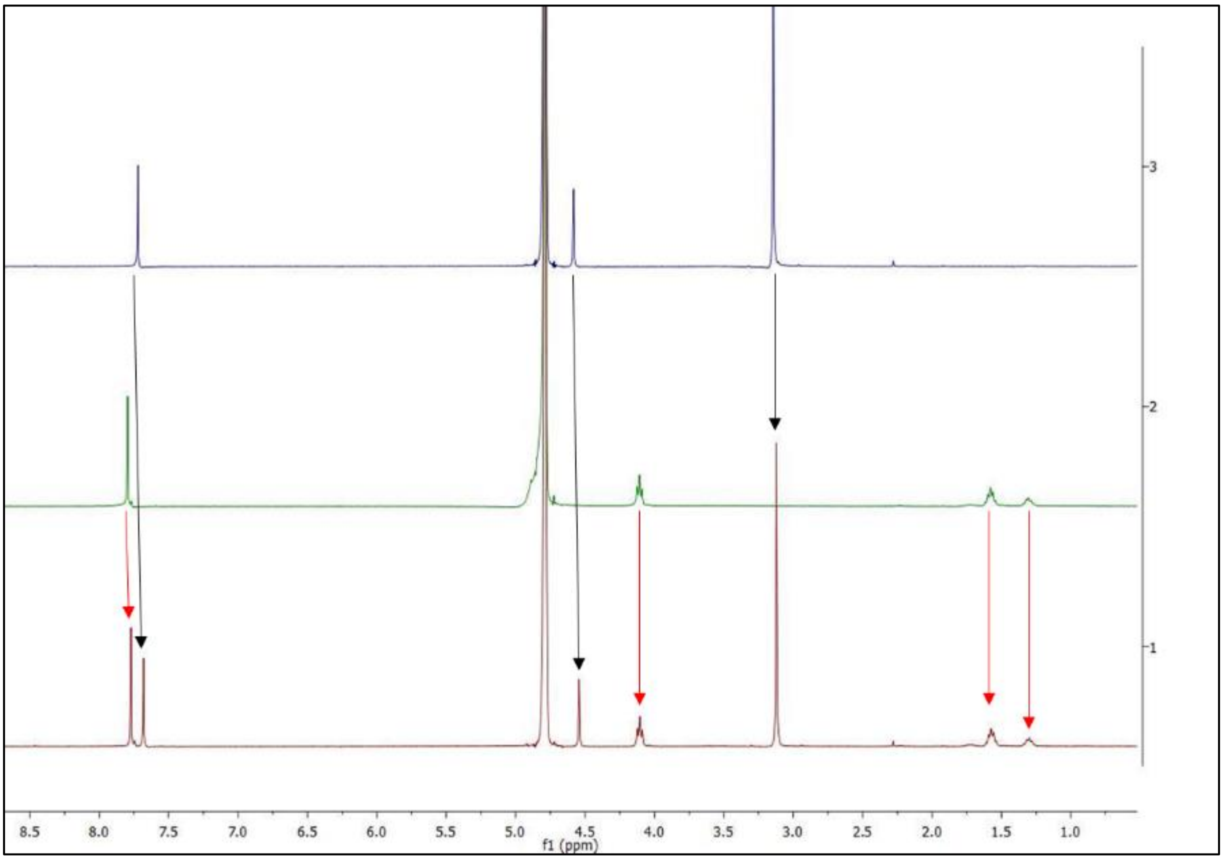

Supplementary Fig. 8a. ^{1}H NMR spectra of **9** (top), **4** (middle) and **9**+**4** (bottom) in D_2O (10^{-3} M, pD 10).

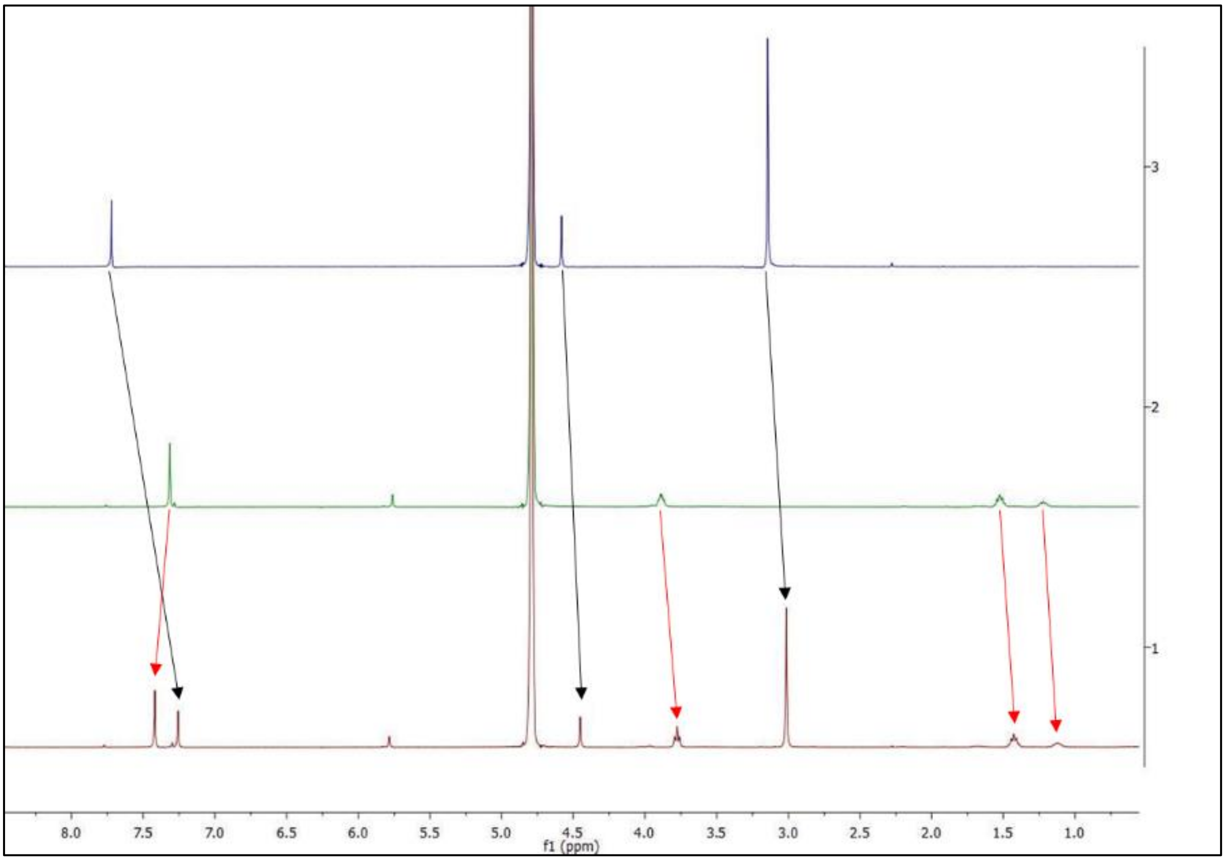

Supplementary Fig. 8b. ^{1}H NMR spectra of **9** (top), **3** (middle) and **9**+**3** (bottom) in D_2O (10^{-3} M, pD 10).

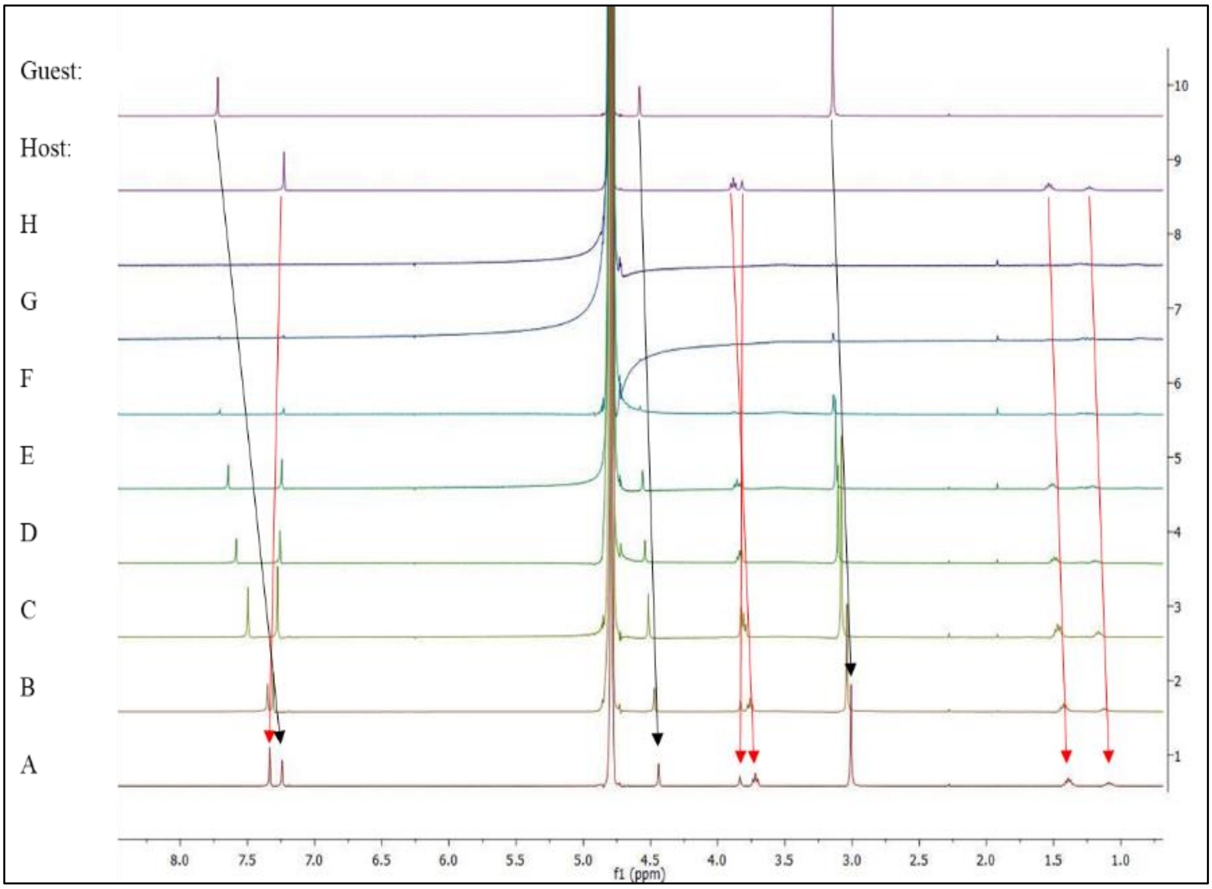

Supplementary Fig. 8c. Dose-dependent ^{1}H NMR spectra. ^{1}H NMR spectra of **9** (Guest), **1** (Host) and **9**+**1** (spectrum A) in D_2O (10^{-3} M each, pD 10). ^{1}H NMR spectra B-H of **9**+**1** at equal concentrations of $5x10^{-4}$, $2x10^{-4}$, 10^{-4}, $5x10^{-5}$, 10^{-5}, $5x10^{-6}$, 10^{-6} M respectively are also given. All spectra are used to determine the binding constant (β) according to the equation[8] $(\Delta\delta/\Delta\delta_{max})/[1 - (\Delta\delta/\Delta\delta_{max})]^2 = \beta a$, where the concentration of host or guest is 'a' and where the binding 1:1 for host and guest. This stoichiometry has been established for closely related systems[2].

Supplementary note 8. Effect of NaCl on the turbidity of mixtures of cyclophanes **1**, **3** or **4** and detergent **10**.

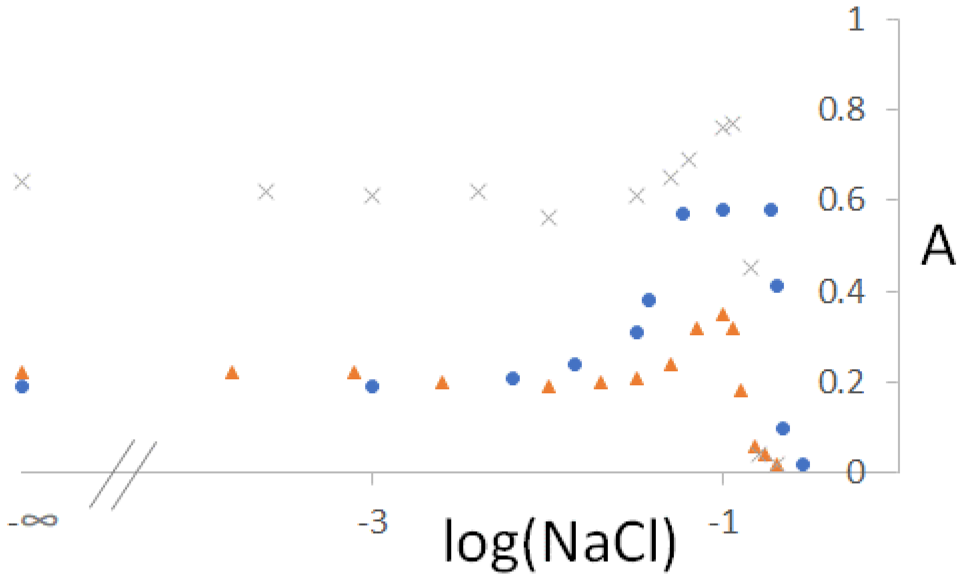

Supplementary Fig. 9. Turbidity (as measured by absorbance (A) at 500 nm with 1 cm path length) versus log(NaCl) for **10** in mixture with **1** (filled circles), **4** (filled triangles) and **3** (crosses) in water.

Supplementary note 9. Logic gate array displayed by the turbidity output of the cyclophane (**1**, **3** or **4**) – detergent **10** system when xylyldiammonium cation **9** is absent.

When **9** is absent, and when only Dialcohol or Diketone can exist at a given moment, i.e. they can't both be present and they can't both be absent, the downstream gate array simplifies first to a XOR and finally to an OR.
The corresponding logic analysis is given below.

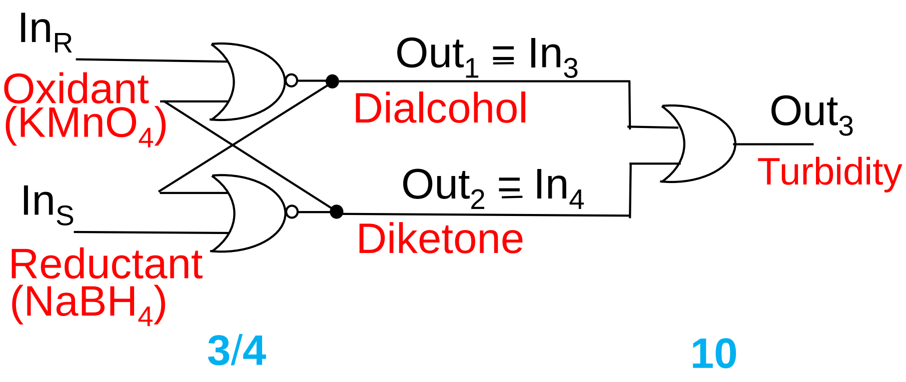

Taking A=In$_3$, B=In$_4$ and C=In$_5$, we get;

$$\begin{aligned} Out_3 &= A\cdot\overline{B}\cdot\overline{C} + \overline{A}\cdot B\cdot\overline{C} + A\cdot\overline{B}\cdot C \\ &= \overline{A}\cdot B\cdot\overline{C} + A\cdot\overline{B}\cdot(C + \overline{C}) \\ &= \overline{A}\cdot B\cdot\overline{C} + A\cdot\overline{B} \end{aligned}$$

If C=0;

$$Out_3 = \overline{A}\cdot B + A\cdot\overline{B}$$

If conditions A=0,B=0 and A=1,B=1 are invalid (since dialcohol or diketone must exist under the experimental milieu);

$$Out_3 = A + B$$

The proof is shown in the truth table below.

A	B	$\overline{A}$	$\overline{B}$	$\overline{A}\cdot B$	$A\cdot\overline{B}$	$\overline{A}\cdot B+A\cdot\overline{B}$	A+B
1	0	0	1	0	1	1	1
0	1	1	0	1	0	1	1

Supplementary references

1. K. Wang, H. -H. Zou, Z. -L. Chen, Z. Zhang, W .-Y. Sun & F. -P. Liang, A series of 3D metal organic frameworks based on [24-MC-6] metallacrown clusters: structure, magnetic and luminescence properties. *Dalton Trans.* 43, 12989-12995 (2014).
2. M. Miyake, M. Kirisawa & K. Koga, Biomimetic studies using artificial systems .6. Design and synthesis of novel cyclophanes having 8 carboxyl groups on the aromatic rings. *Chem. Pharm. Bull.* **41**, 1211-1213 (1993).
3. C. Y. Yao, H. Y. Lin, B. Daly, Y. Xu, W. Singh, H. Q. N. Gunaratne, W. R. Browne, S. E. J. Bell, P. Nockemann, M. Huang, P. Kavanagh & A. P. de Silva, Taming tris(bipyridine)ruthenium(II) and its reactions in water by capture/release with shape-switchable symmetry-matched cyclophanes. *J. Am. Chem. Soc.* **144**, 4977-4988 (2022).
4. B. Daly, T. S. Moody, A. J. M. Huxley, C. Y. Yao, B. Schazmann, A. Alves-Areias, J. F. Malone, H. Q. N. Gunaratne, P. Nockemann & A. P. de Silva, Molecular memory with downstream logic processing exemplified by switchable and self-indicating guest capture and release. *Nat. Commun*. **10**, 49 (2019).
5. M. Miyake, M. Kirisawa & K. Koga, Anionic cyclophanes as hosts for cationic guests. *Tetrahedron Lett*. **32**, 7295-7298 (1991).
6. Phillips, A. P. Synthetic hypotensive agents. II. Some hexamethylene-1,6-bis-t-amines and bis-quaternary salts as ganglionic blocking agents. *J. Am. Chem. Soc.* **77**, 1693–1695 (1955).
7. N. M. van Os, J. R. Haak & L. A. M. Rupert*, Physico-Chemical Properties of Selected Anionic, Cationic and Nonionic Surfactants* (Elsevier Science, Amsterdam, 1993).
8. A. B. Mandal, B. J. Nair & D. Ramaswamy, Determination of the critical micelle concentration of surfactants and the partition coefficient of an electrochemical probe by using cyclic voltammetry. *Langmuir* **4**, 736-739 (1988).
9. A. W. Adamson, *Physical Chemistry of Surfaces,* 5th Edn. (Wiley Interscience, New York, 1990).
10. J. M. Andreas, E. A. Hauser & W. B. Tucker, Boundary tension by pendant drops. *J. Phys. Chem.* **42**, 1001-1019 (1938).
11. K. A. Connors, *Binding Constants: The Measurement of Molecular Complex Stability* (Wiley, New York, 1987).
